# Editorial

**Published:** 2014-01-16

**Authors:** Shibal Bhartiya, Tarek Shaarawy, Tanuj Dada

‘The true method of knowledge is experiment’

—William Blake

The editorial team of the Journal of Current Glaucoma Practice takes great pleasure in bringing to you this issue of the Journal. We hope you will find this issue insightful and relevant to your practice of glaucoma.

In the basic diagnostics section, Sharma et al evaluate the differences between retinal macular thickness and macular volume using optical coherence tomography in primary open angle glaucoma patients and normal subjects. They report a significant difference in the macular parameters as measured on spectral domain-optical coherence tomography (SD-OCT) between the two population subgroups.

Secondary angle closure glaucomas are a distinct entity from primary angle closure glaucoma (PACG). The identifiable contributory factors, therefore, present a great diagnostic and therapeutic challenge for the glaucomatologist. Parivadhini et al review the clinical spectrum of this disease subgroup along with management protocols.

It is well-known that a proportion of eyes will have residual angle closure, despite a patent peripheral iridotomy. In such patients, a laser peripheral iridoplasty may be a useful treatment to ameliorate appositional angle closure that may be occurring through nonpupillary block mechanisms. Leong et al evaluate, by anterior segment optical coherence tomography, the changes in the anterior chamber angle during the short-term postoperative period after laser peripheral iridoplasty.

Lee et al report their initial experiences with the EX-PRESS shunt in a case series of patient with uveitic glaucoma, reporting good intraocular pressure (IOP) control with a propensity for hypotony in the early postoperative period.

In an interesting profile of glaucoma patients from Vietnam, Thanh elucidates the number of antiglaucoma medications required to achieve target pressure and correlates the same to severity of disease.

There has been considerable interest about the potential use of calcium channel blockers in glaucoma therapy, with both ocular hypotensive effects, and increase in perfusion pressure having been attributed to the drug. Ganekal et al evaluate the effect of 0.125% verapamil and 0.5% diltiazem eye drops on IOP in steroid-induced glaucoma in rabbit eyes and report a considerable drop in IOP, equivalent to that due to topical timolol drops.

Kaushik et al report an interesting case of unilateral angle closure glaucoma complicated by an optic disk pit and iridociliary cysts.

As always, we look forward to hearing from you.

Best wishes

**Shibal Bhartiya**

**Tarek Shaarawy**

**Tanuj Dada**

